# Supplementary material for: Inhibition of Liquid–Liquid Phase Separation for Breaking the Solubility Barrier of Amorphous Solid Dispersions to Improve Oral Absorption of Naftopidil
Source: Pharmaceutics. 2022 Nov 30;14(12):2664. doi: 10.3390/pharmaceutics14122664 (PMC9782492; doi:10.3390/pharmaceutics14122664)
Supplement: Supplementary file 1 [file pharmaceutics-14-02664-s001.zip › pharmaceutics-2039699-supplementary.pdf]

Supplementary material File S1

# Inhibition of Liquid-Liquid Phase Separation for Overcoming Dissolution Barrier of Amorphous Solid Dispersions to Improve Oral Absorption of Naftopidil

Masafumi Fukiage[1,*], Kyosuke Suzuki[2], Maki Matsuda[3], Yohei Nishida[4], Michinori Oikawa[5], Takuya Fujita[6], Kohsaku Kawakami[7,*]

[1] Pharmaceutical R&D, Ono Pharmaceutical Co., LTD. 3-3-1, Sakurai, Shimamoto-cho, Mishima-gun, Osaka 618-8585, Japan

[2] Pharmaceutical and ADMET Research Department, Daiichi Sankyo RD Novare Co., Ltd., 1-16-13, Kitakasai, Edogawa-ku, Tokyo 134-8630, Japan

[3] Research & Development Division, Towa Pharmaceutical Co., Ltd., 134, Chudoji Minami-machi, Shimogyo-ku, Kyoto 600-8813, Japan

[4] Technology Research & Development, Sumitomo Dainippon Pharma Co., Ltd., 33-94, Enoki-cho, Suita, Osaka 564-0053, Japan

[5] Pharmaceutical Development Department, Sawai Pharmaceutical Co., Ltd., 5-2-30, Miyahara, Yodogawa-ku, Osaka 532-0003, Japan

[6] College of Pharmaceutical Sciences, Ritsumeikan University, 1-1-1 Noji-Higashi, Kusatsu, Shiga 525-8577, Japan

[7] Research Center for Functionals Materials, National Institute for Materials Science, 1-1 Namiki, Tsukuba, Ibaraki 305-0044

## 1. Physical Characterization of ASDs

X-ray powder diffraction (XRPD) measurements of ASDs were performed on a SmartLab X-RAY diffractometer (Rigaku, Tokyo, Japan). The voltage and the current were 40 kV and 200 mA, respectively. Data were collected at intervals of 0.02º (2 theta) with a scan speed 30º/min.

XRPD measurements proved that absence of diffraction peaks for all ASDs.

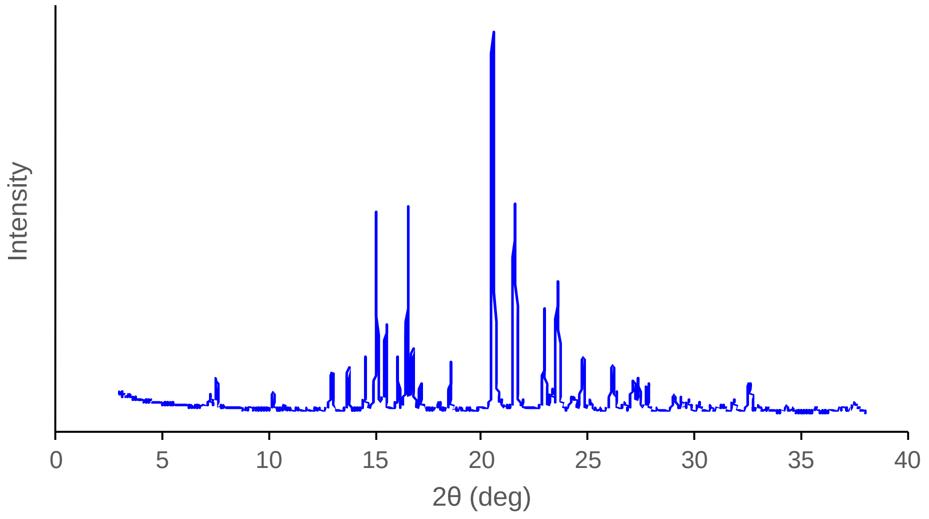


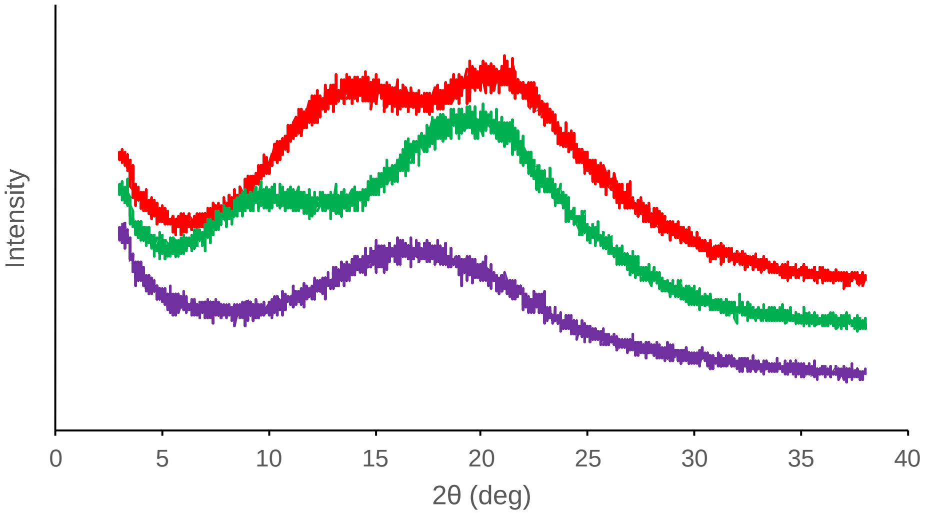

Differential scanning calorimetry (DSC) and temperature-modulated DSC (TM-DSC) were performed on a Discovery DSC (TA Instruments, New Castle, DE, USA), which calibrated monthly using indium and sapphire. Dry nitrogen was used as the inert gas at a flow rate of 50 mL/min. Approximately 2 mg of samples were measured using crimped aluminum pans.

No melting peaks were observed for all the ASDs in the DSC measurements. Single glass transition temperature (Tg) were observed in DSC curves at 82, 85, and 118 °C for PVPVA, HPMCAS, and Eudragit ASDs, respectively. These Tgs were much higher than that for neat NFT glass (25 °C) and in between of those of NFT and polymer, suggesting molecular mixing of NFT and the polymers.

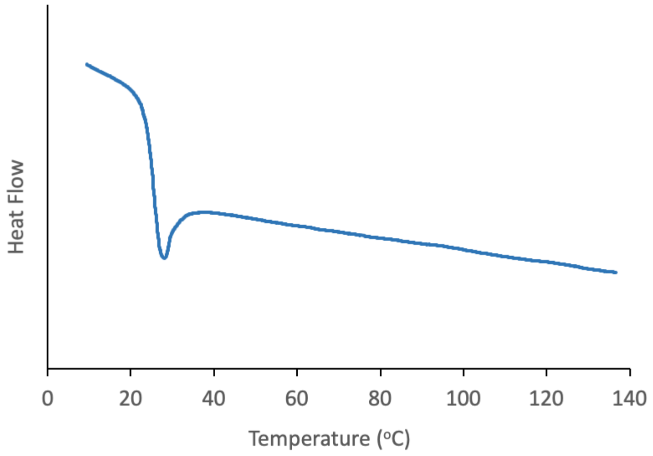


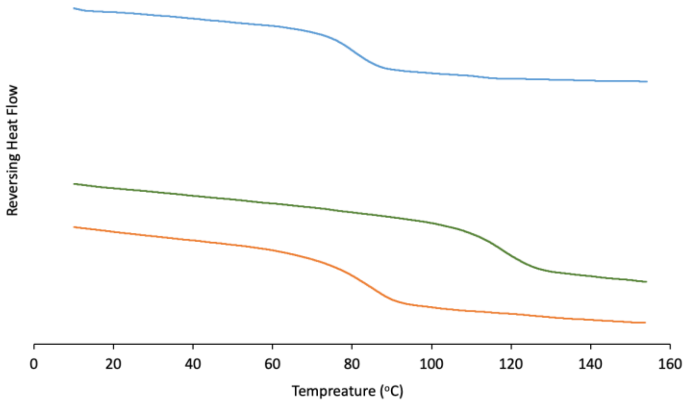

## 2. Detailed numerical data for the D/P/ study.

| | Dose/well (mg) | Apical Solution | Conc. (μg/mL) | | | | | | | | Flux | Papp |
|---|---|---|---|---|---|---|---|---|---|---|---|---|
| | | | Apical | | Apical_free | | Basal | | | | μg/cm2/s | cm/s |
| | | | 15 min | 120 min | 15 min | 120 min | 30 min | 60min | 90min | 120min | | |
| PM | 3.6 | TM | 0.77 | 0.89 | 0.77 | 0.89 | 0.08 | 0.19 | 0.29 | 0.39 | 1.78E-04 | 2.01E-04 |
| HPMCAS ASD | 0.1 | TM | 4.33 | 3.11 | 4.33 | 3.11 | 0.50 | 1.08 | 1.65 | 2.09 | 9.23E-04 | 2.96E-04 |
| | 0.4 | TM | 19.20 | 16.25 | 19.20 | 16.25 | 2.69 | 5.27 | 7.85 | 9.89 | 4.18E-03 | 2.57E-04 |
| | 1.2 | TM | 26.85 | 25.05 | 26.85 | 25.05 | 3.93 | 7.64 | 11.00 | 14.12 | 5.86E-03 | 2.34E-04 |
| | 3.6 | TM | 34.20 | 34.25 | 34.20 | 34.25 | 4.44 | 8.12 | 11.39 | 15.64 | 6.37E-03 | 1.86E-04 |
| Eudragit ASD | 0.1 | TM | 2.04 | 1.33 | 2.04 | 1.33 | 0.34 | 0.74 | 1.12 | 1.40 | 6.10E-04 | 4.61E-04 |
| | 0.4 | TM | 7.65 | 9.95 | 7.65 | 9.95 | 0.93 | 1.89 | 2.96 | 4.27 | 1.91E-03 | 1.92E-04 |
| | 1.2 | TM | 5.00 | 8.00 | 5.00 | 8.00 | 0.64 | 1.29 | 2.06 | 2.84 | 1.27E-03 | 1.59E-04 |
| | 3.6 | TM | 1.94 | 2.44 | 1.94 | 2.44 | 0.28 | 0.54 | 0.79 | 1.01 | 4.26E-04 | 1.75E-04 |
| PM | 3.6 | SIF | 23.31 | 24.66 | 1.47 | 1.56 | 0.26 | 0.55 | 0.84 | 1.13 | 5.00E-04 | 2.03E-05 |
| HPMCAS ASD | 0.1 | SIF | 7.19 | 7.99 | 0.45 | 0.50 | 0.08 | 0.22 | 0.36 | 0.49 | 2.34E-04 | 2.94E-05 |
| | 0.4 | SIF | 34.35 | 40.25 | 2.17 | 2.54 | 0.32 | 0.73 | 1.20 | 1.61 | 7.45E-04 | 1.85E-05 |
| | 1.2 | SIF | 105.60 | 119.55 | 6.66 | 7.54 | 0.87 | 1.61 | 2.90 | 3.97 | 1.83E-03 | 1.53E-05 |
| | 3.6 | SIF | 425.70 | 443.70 | 26.86 | 27.99 | 1.83 | 4.61 | 7.53 | 9.88 | 4.67E-03 | 1.05E-05 |
| Eudragit ASD | 0.1 | SIF | 7.24 | 7.75 | 0.46 | 0.49 | 0.10 | 0.24 | 0.36 | 0.48 | 2.18E-04 | 2.82E-05 |
| | 0.4 | SIF | 29.55 | 34.95 | 1.86 | 2.20 | 0.29 | 0.71 | 0.99 | 1.40 | 6.22E-04 | 1.78E-05 |
| | 1.2 | SIF | 73.35 | 101.85 | 4.63 | 6.43 | 0.43 | 1.01 | 1.55 | 2.40 | 1.11E-03 | 1.09E-05 |
| | 3.6 | SIF | 48.15 | 58.50 | 3.04 | 3.69 | 0.26 | 0.58 | 0.80 | 1.02 | 4.29E-04 | 7.34E-06 |
